# Macular morphology after cataract surgery with and without primary posterior continuous curvilinear capsulorhexis

**DOI:** 10.3389/fmed.2025.1687460

**Published:** 2025-10-28

**Authors:** Chishan Kang, Kunxia Lin, Yulong Huang, Mengting Yu, Wenjie Wu

**Affiliations:** ^1^Shengli Clinical Medical College of Fujian Medical University, Fujian Medical University, Fuzhou, China; ^2^Department of Ophthalmology, Fujian Provincial Hospital, Fuzhou University Affiliated Provincial Hospital, Fuzhou, China

**Keywords:** cystoid macular edema, posterior vitreous detachment (PVD), cataract surgery, posterior capsulorhexis, surgery technique

## Abstract

**Purpose:**

To evaluate changes in macular morphology after cataract surgery with and without primary posterior continuous curvilinear capsulorhexis (PPCCC).

**Methods:**

A prospective, intraindividual, randomized clinical trial was performed at Fuzhou University Affiliated Provincial Hospital, Fujian, China. A total of 130 eyes of 65 age-related cataract patients with normal macular morphology and function waiting for bilateral cataract surgery and intraocular lens (IOL) implantation were enrolled. Cataract surgery combined with PPCCC was performed in one eye, and routine cataract surgery in the fellow eye (NPCCC group). Optical coherence tomography (OCT) measurements were performed in all patients preoperatively and postoperatively on 1 day, 1 week, 1 month, and 3 months.

**Results:**

A total of 120 eyes of 60 patients were capable to complete scheduled follow-ups and analyzed in the study. There was no statistically significant difference between the PPCCC group and NPCCC group in terms of subfoveal central retinal thickness (CRT), central 1-mm subfield (CSF), average retinal thickness in the middle (1–3 mm) and outer (3–6 mm) rings (*p* > 0.05) at all timepoints after surgery. Three eyes developed cystoid macular edema (CME) 1-month post-surgery. One eye in the PPCCC group recovered in 2 weeks after topical treatment, while two in the NPCCC group took 8 weeks to recover. In the NPCCC group, PVD progressed in two eyes, one from stage 2 to 4, and another from stage 1 to 2. No PVD progression in the PPCCC group. The corrected distance visual acuity (CDVA) of all patients was logMAR 0.1 or better at the last visit.

**Conclusion:**

Cataract surgery with combined manual PPCCC does not increase the risk of CME and PVD in patients. PPCCC is a safe cataract surgery technique.

## Introduction

Significant advancements in adult cataract surgery techniques and equipment have markedly improved postoperative refractive outcomes and patients’ satisfaction (1). However, posterior capsular opacification (PCO) persists as the predominant complication following cataract surgery, obscuring the optic zone, and precipitating a decline in visual acuity (2). Moreover, the uneven migration and proliferation of lens epithelial cells (LECs) inside capsular bag may lead to secondary intraocular lens (IOL) tilt, decentration, and even rotation especially affecting premium IOLs (3, 4). The primary treatment to PCO is neodymium: yttrium–aluminum–garnet (Nd:YAG) laser capsulotomy, while it might damage the anterior hyaloid and carry potential risks such as cystoid macular edema (CME), spikes in intraocular pressure, and retinal detachment (5).

Recently, many researchers are actively focused on developing effective prevention and treatment strategies for PCO (6–12). Among them, primary posterior continuous curvilinear capsulorrhexis (PPCCC) in adult cataract surgery has been gaining notable attention (7–12). It is reported that PPCCC has promising potential to significantly delay PCO and reduce the necessity for Nd:YAG laser capsulotomy (10, 11). Prior researchers have applied PPCCC on more than 1,000 patients and achieved a low rate of postoperative complications, less axial movement, and better centration of IOLs (12–15). Besides, our previous studies have proved that PPCCC brings faster posterior capsule adhesion to IOL leading to better centration and stability of IOLs (7, 9, 16). Despite its advantages, surgeons may still be concerned about the safety of this technique, although the PPCCC technique removes only the posterior capsule and retains the anterior hyaloid membrane intact, some researchers are still concerned about its potential additional effects on the anterior and posterior segments (11, 14).

Previous studies have shown that PPCCC and non-PPCCC (NPCCC) have similar anterior segment safety, with comparable levels of anterior chamber flare, cell debris, and intraocular pressure (14, 15, 17). In addition, several investigators provided valuable insights into the posterior segment safety profile of PPCCC, particularly regarding its effect on the macula (10, 11, 18, 19). Stifter et al. (11) and Yazici et al. (18) reported no case of CME following PPCCC. However, these two studies were limited by time domain OCT (TD-OCT), which offered inferior resolution and detection capabilities than spectral-domain OCT (SD-OCT). Al-Nashar and Khalil (10) identified two cases of CME among 25 patients with SD-OCT, suggesting that PPCCC would not significantly elevate the risk of CME, but the study was case series with limited sample size and lack of comparative control groups. Besides, incomplete posterior vitreous detachment (PVD) is known to potentially cause vitreomacular traction syndrome, a risk factor for CME (20, 21). However, past research seldom focused on the occurrence and progression of PVD after cataract surgery combined with PPCCC.

To the best of our knowledge, no preceding study has assessed the impact on macular and PVD of manual PPCCC using SD-OCT with a rigorous design. Thus, we conducted a prospective intraindividual randomized controlled trial to evaluate macular morphology changes in patients with normal macula, providing reliable safety data for PPCCC.

## Methods

### Participants

**Figure 1:**
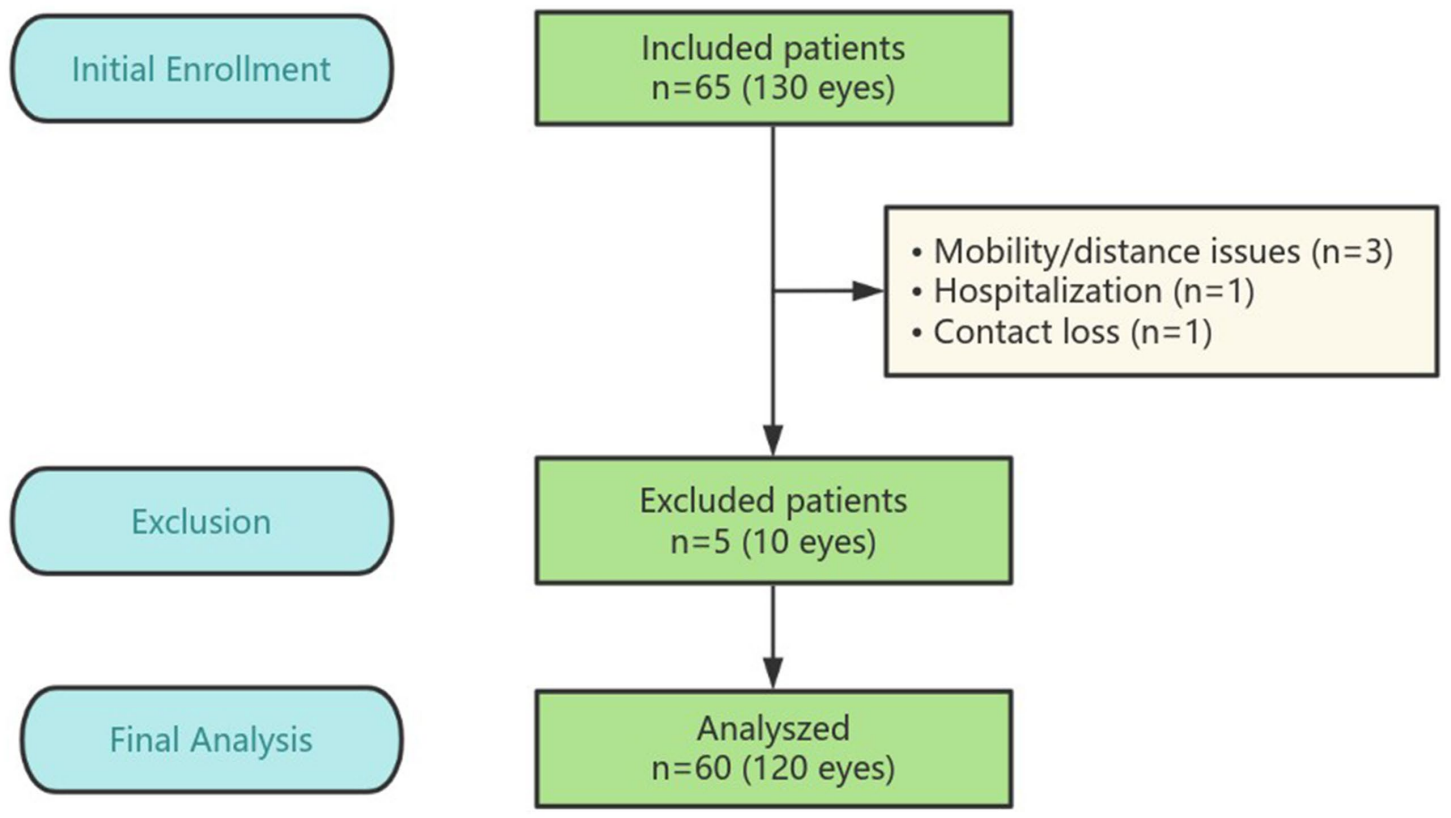
The study flowchart.

The prospective, intraindividual, comparative randomized controlled trial (RCT) included 130 eyes from 65 patients with age-related cataract who underwent bilateral cataract surgery at Fuzhou University Affiliated Provincial Hospital in Fujian, China, between December 2023 and May 2024 (flowchart details in Figure 1). The study protocol was approved by the institutional review board of Fuzhou University Affiliated Provincial Hospital and adhered to the ethical standards of the Declaration of Helsinki. All potential participants were thoroughly informed about the potential benefits and risks associated with the study. In compliance with the Declaration of Helsinki, written informed consent was obtained from each participant. The clinical trial is registered with the number ChiCTR2300078457.

The inclusion criteria were: (1) a diagnosis of bilateral age-related cataract; and (2) a planned interval of less than 2 weeks between surgeries for both eyes. The exclusion criteria were: (1) pre-existing retinal or macular conditions such as epiretinal membrane, diabetic retinopathy (DR), macular hole, age-related macular degeneration, or retinal vascular disease; (2) pre-existing ocular conditions such as glaucoma, uveitis, or eye trauma; (3) previous intraocular surgery or laser treatment; (4) use of topical medications; and (5) poor-quality or unreliable OCT images obtained preoperatively;(6) anisometropia. Patients who experienced intraoperative complications, such as iatrogenic posterior capsule rupture or tear, or those with obvious posterior capsular plaques, were excluded from both groups.

Before enrollment, all patients underwent a series of preoperative ophthalmologic examinations, including uncorrected distance visual acuity (UDVA; logMAR), anterior chamber depth (ACD), axial length (AL), slit lamp microscopy, intraocular pressure (IOP) measured by non-contact tonometry, optical biometry, and fundus examination with OCT (Heidelberg Spectralis version 1.10.0.0, Germany). Postoperatively, all patients were scheduled for routine follow-up at 1 day, 1 week, 1 month, and 3 months. The follow-up examinations included UDVA, corrected distance visual acuity (CDVA), binocular automatic refractometry (Auto Refractometer AR-610, Nidek Co., Japan), slit lamp microscopy, and fundus examination with Spectralis OCT.

### Randomization

Before the operation, the data researcher randomly selected one of the two identical envelopes containing the PPCCC or NPCCC allocation to determine the surgical method of the first operated eye, and the surgical method of the contralateral eye was then determined. Cataract surgery with combined PPCCC was performed in one eye, and routine cataract surgery in the contralateral eye (NPCCC group) and the posterior lens capsule kept untouched. Throughout the study, treatment assignment is confidential to the patients and the investigator, who in charge of the examination, while group assignment was masked to the surgeon until the surgery commenced. The surgical procedures and the postoperative medication were standardized in all patients.

### Surgical technique

The same experienced surgeon (WJ.W.) performed all cataract surgeries using the surgical procedure that has been described previously (8). A temporal 2.4 mm clear corneal incision was created. Sodium hyaluronate 15 mg/mL (Shanghai Qisheng Biological Preparation Co., Ltd.) was used as the ophthalmic viscosurgical device (OVD). Nuclear removal, cortical aspiration, and posterior capsular polishing were performed within a well-centered 5.5 mm anterior continuous curvilinear capsulorhexis, and subsequent procedures were performed according to group allocation.

In the PPCCC group, the posterior capsule center was punctured using a 22-gauge needle to create an approximately 2 mm fissure. Then, the OVD was injected into the capsular bag. Following the outlines of the anterior continuous curvilinear capsulorhexis (ACCC), a well-centered about 4.0 mm PPCCC was created. After removing the central capsule flap, a 1-piece 360-degree square-edge acrylic TECNIS^®^ IOL (Johnson & Johnson Vision, United States) was then inserted into the bag. Ultimately, the residual OVD was aspirated including that posterior to the IOL and the surgical wounds were then watertight.

### Postoperative medication

All patients were administered Tobramycin and Dexamethasone Eye Drops (Novartis, Belgium, 15 mg: 5 mg/5 mL) four times daily, with a gradual reduction by one increment every 5 days until discontinuation after 20 days. Carbomer Eye Gel (Bausch & Lomb, Germany, 10 g: 20 mg/10 g) was applied three times daily until the gel ran out. Pranoprofen Eye Drops (Senju, Japan, 0.1%/5 mL) were started three times daily from 2 weeks postoperatively until the bottle was finished. Each medication was instilled 5 min apart, with one drop at a time. Both eyes received the same postoperative therapy.

### Optical coherence tomography measurements

**Figure 2:**
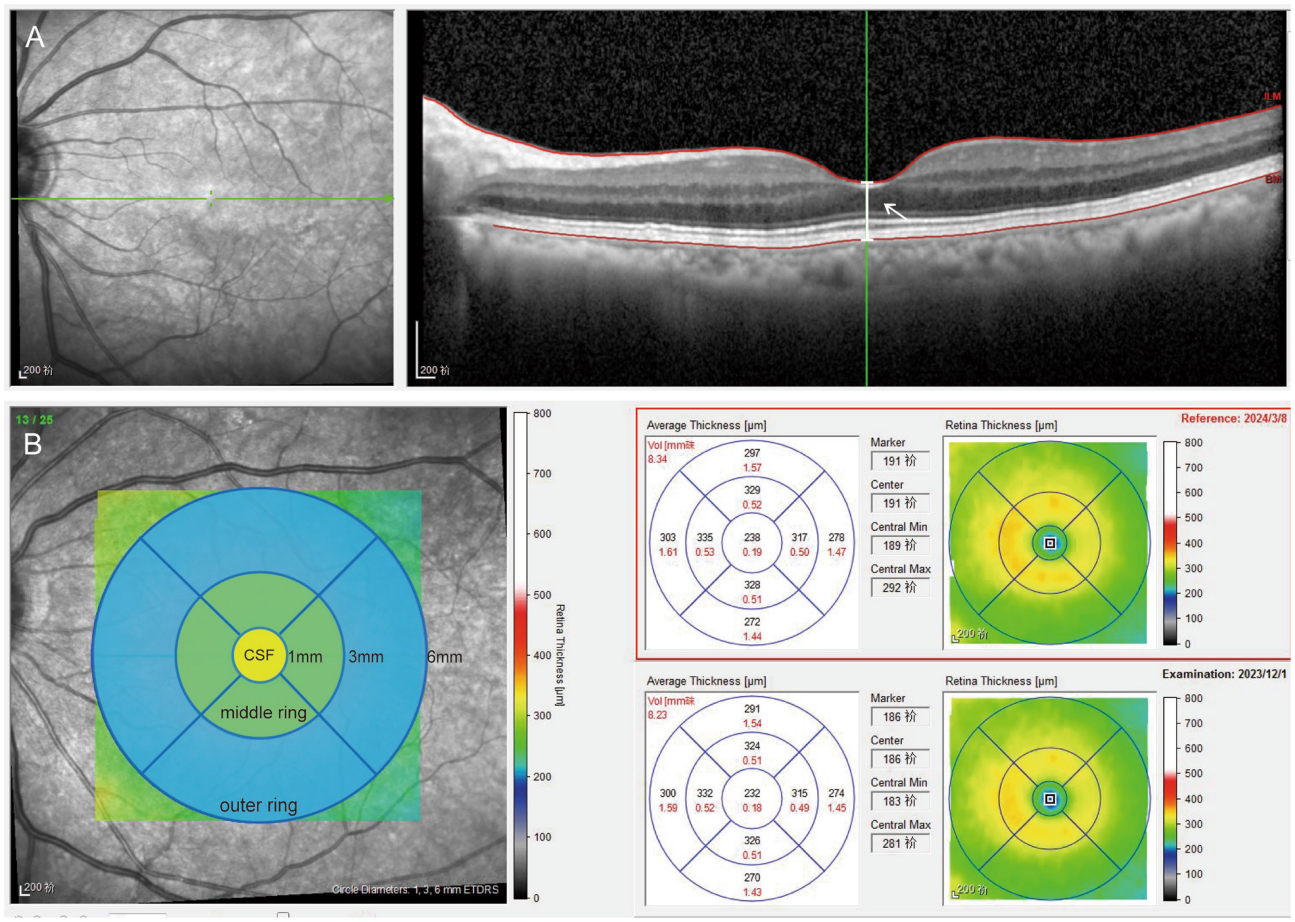
Picture **A** shows a horizontal raster SD OCT scan, the white arrow points the white line means the distance between the vitreo-retinal interface and the RPE-Bruch membrane junction at the center of the foveal depression. Picture **B** shows the retinal map concluding 1 mm central subfield and the 1–3 mm middle and 3–6 mm outer rings. The retinal thickness of the rings was evaluated in the four quadrants (inferior, superior, nasal, and temporal). The yellow circle means 1-mm central subfield (CSF). The green circle means the average values of the four quadrants of 1–3 mm middle ring. The blue circle means the average values of the four quadrants of 3–6 mm outer ring.

The OCT examination was performed by an experienced operator. All scans were acquired in a dark room without mydriasis. The standard imaging protocol is as follows: A horizontal raster SD OCT scan of 20 × 20° was taken through the foveal center, consisting of 25 sections with an automatic real-time (ART) setting of 9 (averaging 9 images). Preoperative scans were marked as the patient’s baseline and were used for referencing subsequent scans using the “follow-up” function, ensuring that the scans were performed at the same location. The scans were conducted postoperatively on 1 day, 1 week, 1 month, and 3 months (see Figure 2).

Macular measurements were performed using the inbuilt Spectralis mapping software, Heidelberg Eye Explorer (version 6.0c). (1) Subfoveal central retinal thickness (CRT), which represents the distance between the vitreo-retinal interface and the RPE-Bruch membrane junction at the center of the foveal depression (Figure 2). (2) The area values were extrapolated from the retinal map: (1) 1-mm inner ring: central 1-mm subfield (CSF), the mean retinal thickness within the central 1-mm diameter area; (2) 1–3 mm middle ring: mean retinal thickness in 1–3 mm area; (3) 3–6 mm outer ring: mean retinal thickness in 3–6 mm area, with the average value automatically calculated for all four quadrants. The average values of the four quadrants were used for data analysis (Figure 2). All B-scan images were reviewed for potential errors in automatic segmentation, and all OCT thickness measurements were manually verified by two experienced operators to avoid quantitative errors.

### Diagnosis of CME

The diagnosis of cystoid macular edema (CME) was defined as a 30% increase in baseline CRT and/or macular thickening associated with definite cystic changes detected by OCT (22, 23). If CME was detected by OCT, pranoprofen (a nonsteroidal anti-inflammatory drug) and prednisolone acetate (a steroidal drug) were administered four times a day, with an additional follow-up OCT scheduled after 2 weeks of treatment.

### Statistical analysis

The sample size was calculated using PASS 15.0. Based on historical data indicating a CME incidence of 0.1–30% (24) in the control group and 0–8% (10) in the intervention group, the sample size estimation assumed a two-sided significance level (*α* = 0.05) and 80% statistical power (1 − *β* = 0.80). Using the formula for comparing two independent proportions, the calculation yielded approximately 46 eyes per group. To account for a potential attrition rate of 10% and ensure robustness, the target sample size was increased to 65 eyes per group. Descriptive data are presented as the mean ± standard deviation (SD). If the data were normally distributed, between-group differences were determined using the paired *t*-test. If the data were not normally distributed, the Mann–Whitney rank-sum test was performed. Pearson correlation coefficients were calculated for normally distributed data, while Spearman correlation coefficients were used for non-normally distributed data. Repeated-measures analysis of variance (ANOVA) was used to compare clinical conditions within individual participants at different time points. All statistical analyses were performed using SPSS (v. 24, SPSS, Inc.). Differences with a *p*-value <0.05 were considered statistically significant.

## Results

### Demographic data

After follow-up for 3 months, five patients did not complete their scheduled follow-ups: three missed the 3-month follow-up due to mobility issues or distance inconvenience, one was hospitalized and unable to attend, and one is unreachable, possibly due to a changed contact number. In total, 120 eyes of 60 patients were analyzed in the study, the mean age is 69.17 ± 7.30 years (range, 50 to 85 years). 20 (33.3%) were male and 40 (66.7%) were female. There are 12 patients with DM and 48 patients without DM. Patient demographic characteristics are depicted in Table 1, there was no significant difference between the two groups in anterior chamber depth (ACD), axial length (AL), intraocular pressure (IOP) and preoperative CDVA (*p* > 0.05, Mann–Whitney *U* test). And no difference was found between the two groups in CRT, CSF, averaged retinal thickness of 1–3 mm and 3–6 mm area preoperatively (*p* > 0.05, paired *t*-test). There are no intraoperative complications such as iatrogenic posterior capsule rupture or tear occurred in any patient of both groups.

**Table 1:** Demographic data.

Parameter	PPCCC group (*N* = 60)	NPCCC group (*N* = 60)	*p*-value
Eye (right/left)	60/60		
Gender (male/female)	20/40		
Diabetes mellitus (with/without)	12/48		
Age (y)	69.17 ± 7.30		
ACD (mm)	3.074 ± 0.469	3.044 ± 0.459	0.922
AL (mm)	23.88 ± 1.94	23.82 ± 1.89	0.846
IOP (mm Hg)	16.23 ± 2.48	16.24 ± 2.87	0.986
Baseline CDVA (log MAR)	0.589 ± 0.301	0.558 ± 0.319	0.588
CRT (μm)	218.88 ± 15.049	217.10 ± 15.430	0.523
CSF (μm)	253.08 ± 13.217	251.70 ± 14.105	0.580
3 mm-area (μm)	323.31 ± 14.079	323.07 ± 14.477	0.926
6 mm-area (μm)	287.85 ± 14.514	286.27 ± 14.605	0.552

PPCCC, primary posterior continuous curvilinear capsulorhexis; NPCCC, no posterior continuous curvilinear capsulorhexis; ACD, anterior chamber depth; AL, axial length; IOP, intraocular pressure; Baseline CDVA, baseline of correct distance visual acuity; CRT, subfoveal central retinal thickness; CSF, central 1-mm subfield; 1–3 mm area, mean retinal thickness in 1–3 mm middle ring; 3–6 mm area, mean retinal thickness in 3–6 mm outer ring.

The eyes included in the study were divided into two distinct subgroups based on the presence of DM, and further categorized into five subgroups according to the preoperative stage of PVD.

### Visual acuity

**Figure 3:**
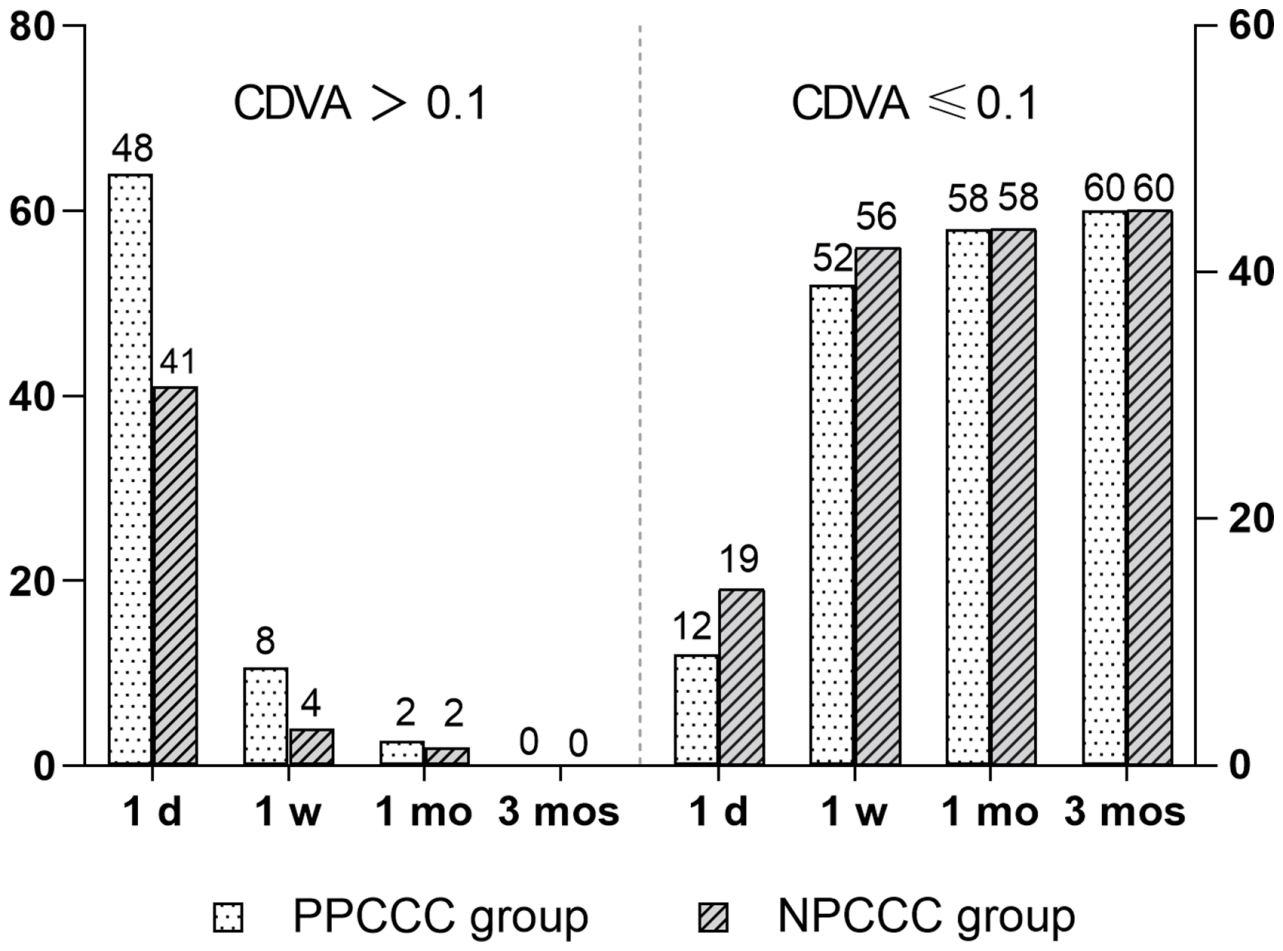
Changes in corrected distance visual acuity (CDVA, logMAR) in the PPCCC and NPCCC groups at 1-day, 1-week, 1-month, and 3-month follow-ups after surgery. CDVA > 0.1: visual acuity worse than 0.1 logMAR. CDVA ≤ 0.1: visual acuity of 0.1 logMAR or better.

Figure 3 showed the number of patients with visual acuity less than 0.1 CDVA (logMAR) gradually increased, over time postoperatively. And all patients in both groups had the CDVA of 0.10 or better at last follow up. After a three-month postoperative period, there was a marked improvement in CDVA from 0.59 ± 0.30 preoperatively to 0.02 ± 0.04 (*p* < 0.001) in PPCCC group, and 0.56 ± 0.32 to 0.01 ± 0.03 (*p* < 0.001) in NPCCC group (Table 2). There was no significant difference in CDVA between the two groups at any visit (Table 2).

**Table 2:** CDVA, CRT, CSF, averaged retinal thickness of 1–3 mm middle and 3–6 mm outer rings.

Parameters	PPCCC group	NPCCC group	*p*-value
CDVA (logMAR)			
1 day	0.2 ± 0.18	0.23 ± 0.16	0.23
1 week	0.06 ± 0.08	0.05 ± 0.07	0.49
1 month	0.03 ± 0.06	0.03 ± 0.06	0.75
3 months	0.02 ± 0.04	0.01 ± 0.03	0.63
CRT			
1 day	215.8 ± 15.8	216.2 ± 16.0	0.90
1 week	213.2 ± 15.7	215.4 ± 16.6	0.46
1 month	222.6 ± 21.8	227.2 ± 25.9	0.29
3 months	222.0 ± 17.3	223.9 ± 16.5	0.54
CSF			
1 day	250.2 ± 13.2	250.98 ± 15.3	0.75
1 week	251.6 ± 14.6	251.8 ± 16.4	0.95
1 month	262.5 ± 19.8	264.8 ± 26.7	0.59
3 months	258.8 ± 16.3	259.1 ± 17.8	0.92
1–3 mm area			
1 day	320.1 ± 13.3	321.3 ± 14.0	0.61
1 week	325.7 ± 13.5	325.7 ± 15.4	0.97
1 month	330.8 ± 15.7	332.1 ± 19.3	0.69
3 months	330.4 ± 13.5	331.8 ± 15.0	0.90
3–6 mm area			
1 day	285.0 ± 14.5	285.9 ± 14.2	0.75
1 week	288.8 ± 13.7	288.3 ± 14.5	0.86
1 month	293.9 ± 15.0	294.6 ± 15.3	0.82
3 months	293.7 ± 14.3	292.7 ± 15.7	0.87

The CDVA, CRT, CSF, averaged retinal thickness of 1–3 mm middle and 3–6 mm outer rings change between PPCCC group and NPCCC group at 1 day, 1 week, 1 month, and 3 months postoperatively.

### Retinal thickness

**Figure 4:**
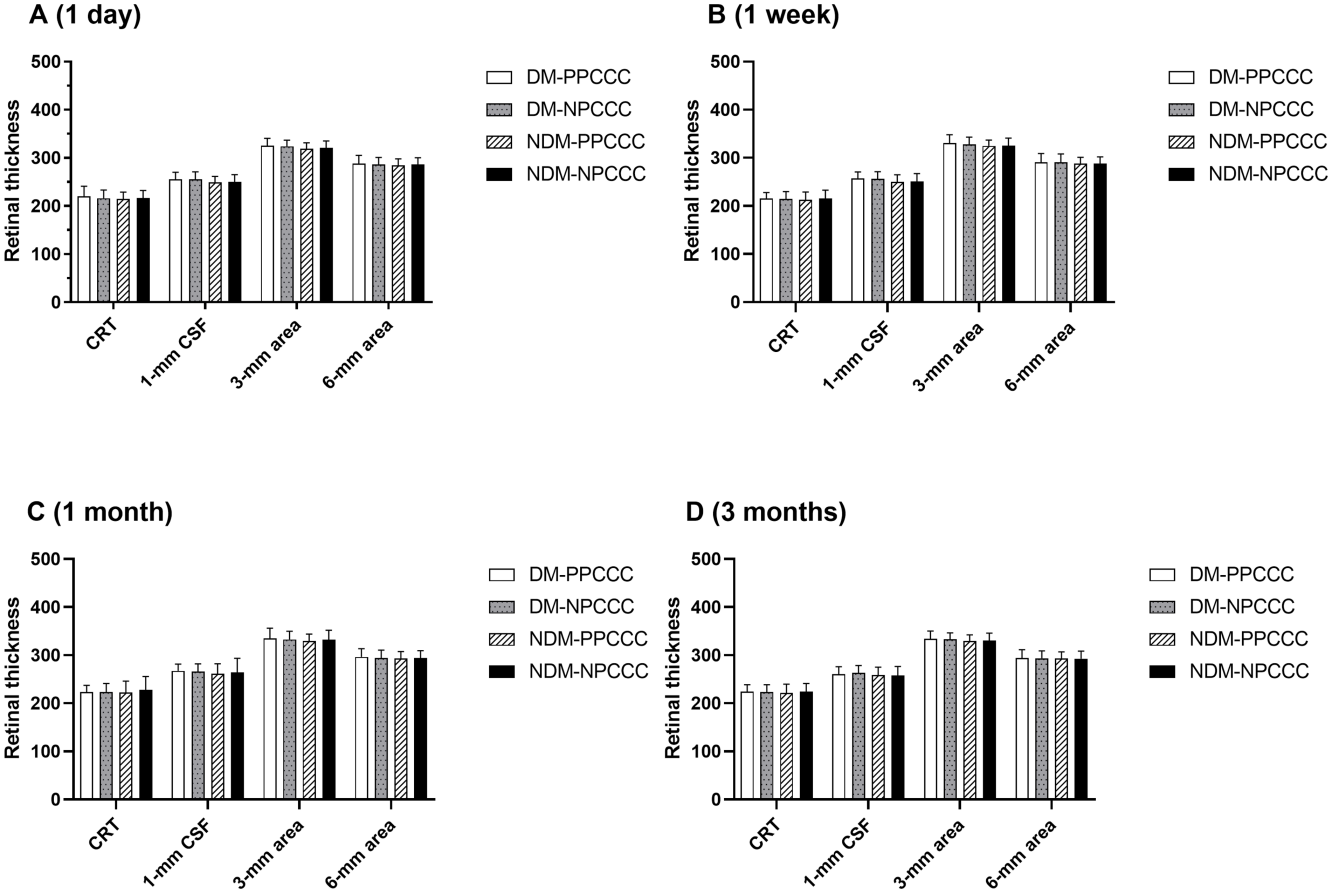
Retinal thickness comparisons between patients with diabetes mellitus (DM) and non-diabetic patients in the PPCCC and NPCCC groups at 1-day, 1-week, 1-month, and 3-month follow-ups. CRT, subfoveal central retinal thickness; DM-PPCCC: diabetes mellitus patients in PPCCC group; NDM-PPCCC: non-diabetic patients in PPCCC group: DM-NPCCC: diabetes mellitus patients in NPCCC group; NDM-NPCCC: non-diabetic patients in NPCCC group: CSF, central 1-mm subfield; 1–3 mm area, mean retinal thickness in 1–3 mm middle ring; 3–6 mm area, mean retinal thickness in 3–6 mm outer ring.

There was no difference between the two groups in CDVA, CRT, CSF, averaged retinal thickness of 1–3 mm and 3–6 mm area during scheduled follow-up. Figure 4 demonstrated the changes in retinal thickness of patients with or without DM in PPCCC and NPCCC group. No significant change in retinal thickness was found between PPCCC group and NPCCC group in either DM or non-DM patients at all postoperative visits (*p* > 0.05, repeated measures ANOVA).

### Cystoid macular edema

**Figure 5:**
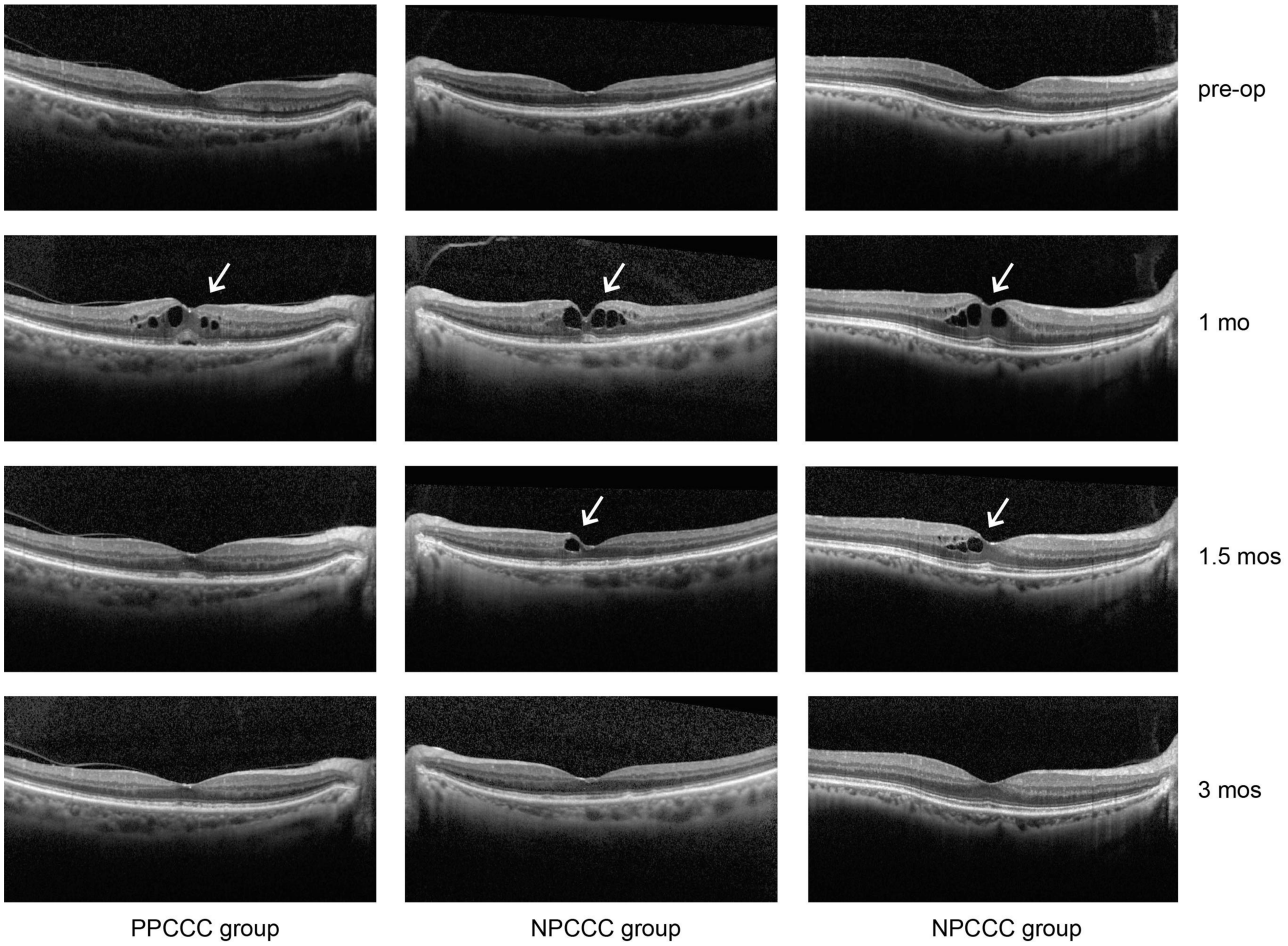
Cystoid macular edema (CME) in three patients from the PPCCC and NPCCC groups, indicated by white arrows on spectral-domain optical coherence tomography (SD-OCT). All cases occurred at 1-month postoperatively. Pre-op, preoperative; 1 mo, 1 month; 1.5 mos, 1.5 months; 3 mos, 3 months.

No cases of clinically significant CME were observed, but CME was observed in three eyes of two patients (2.5% of eyes) (two females, one is 74 years old, and the other is 85) without visual impairment occurring 1 month postoperatively. Neither patient had a history of DM, and both are female. After application of pranoprofen and prednisolone acetate, one eye (PPCCC group) returned to normal with 2 weeks, and two eyes (NPCCC group) recovered 8 weeks (Figure 5).

### Occurrence and progression of PVD

**Figure 6:**
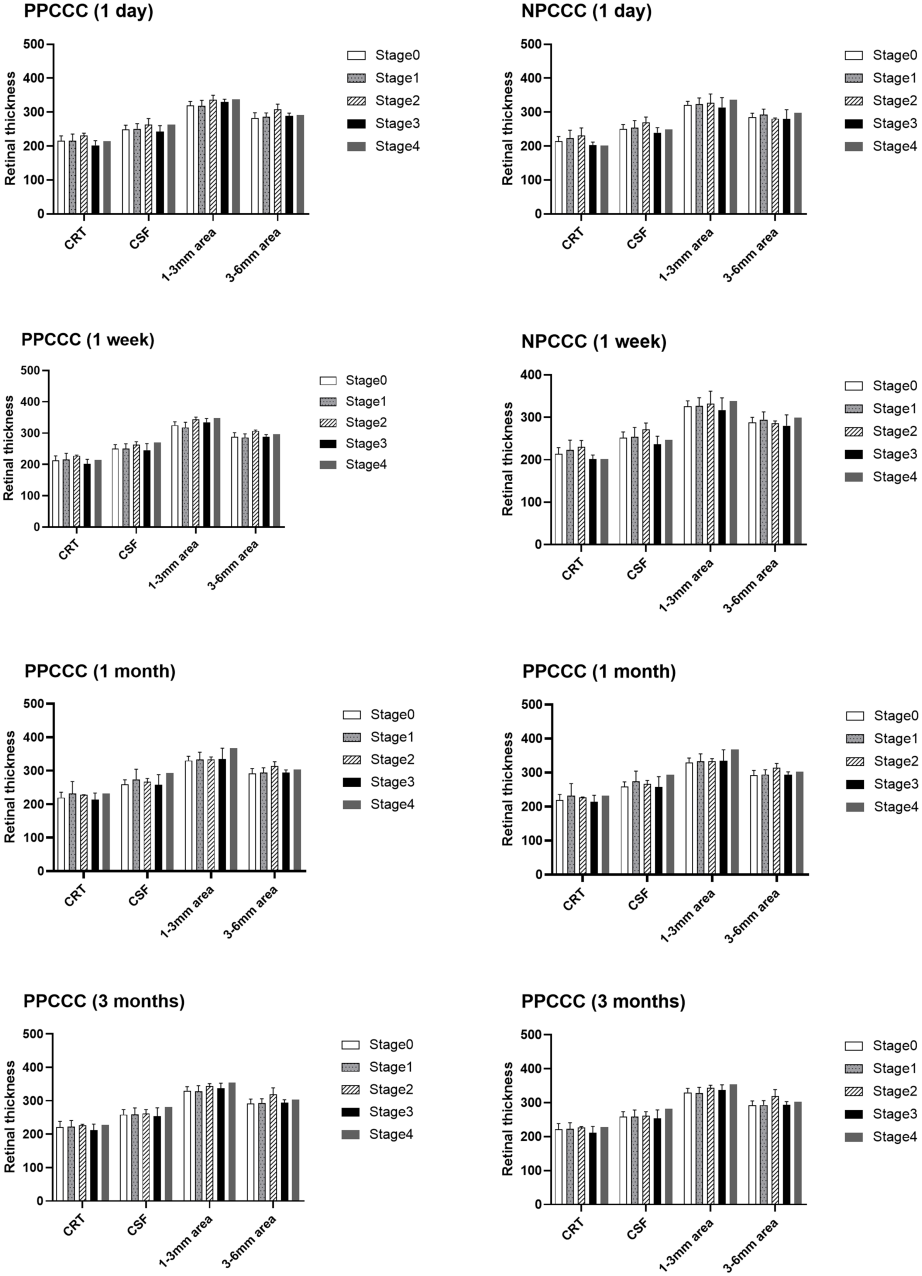
Retinal thickness across posterior vitreous detachment (PVD) stages at 1-day, 1-week, 1-month, and 3-month postoperative follow-ups.

**Figure 7:**
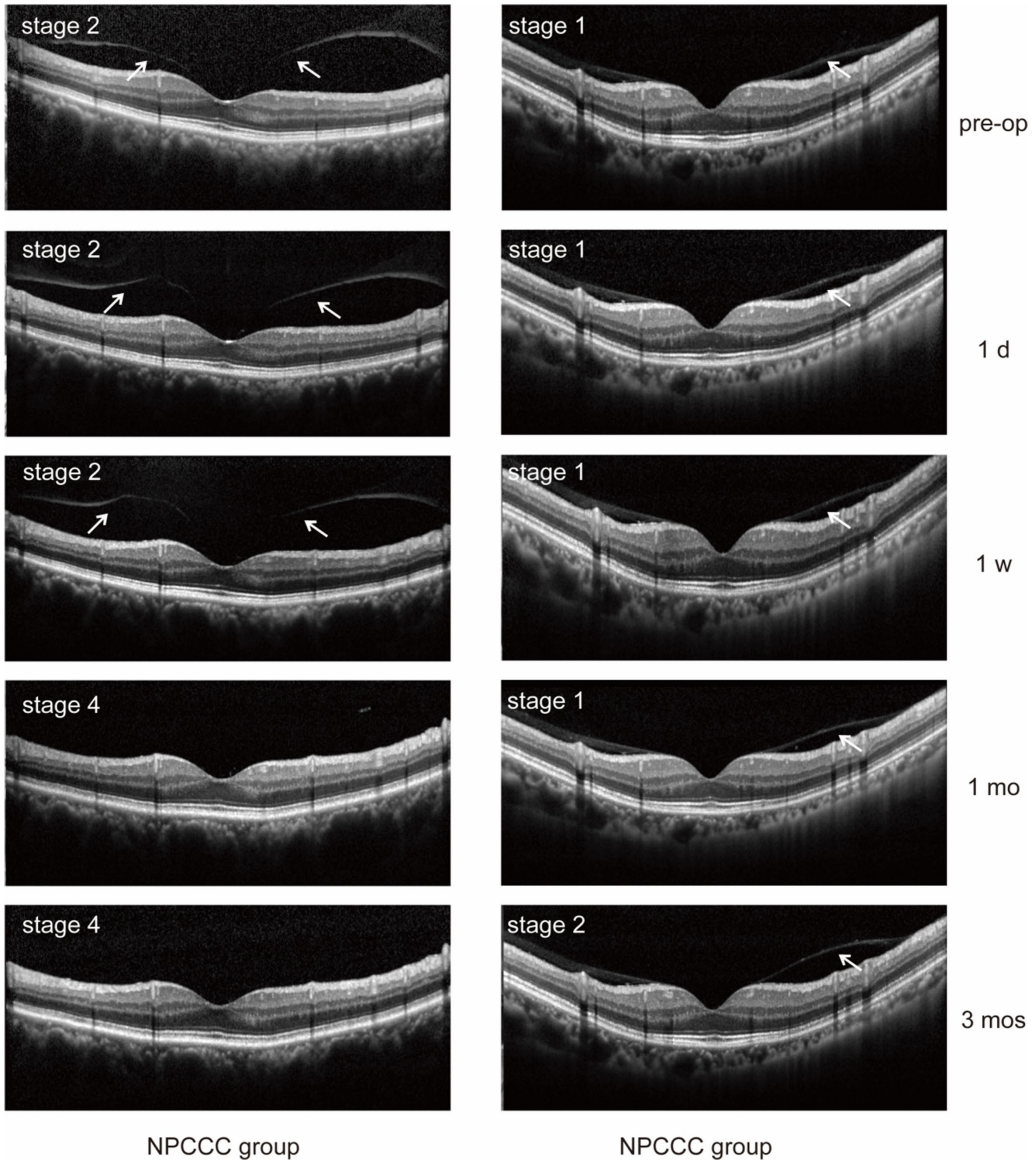
Posterior vitreous detachment (PVD) progression was observed in two eyes in the NPCCC group: one eye progressed from stage 2 to 4, and another from stage 1 to 2. No PVD progression occurred in the PPCCC group.

We excluded three eyes before surgery due to excessive noise impedes the observation of PVD. Table 3 illustrated the comparative changes in the number of patients at various stages of PVD preoperatively and at 1 day, 1 week, 1 month, and 3 months postoperatively between the two study groups. No significant difference in PVD occurrence across five stages was found between the PPCCC and NPCCC groups preoperatively or at any follow-up visit (*p* > 0.05, repeated measures ANOVA, Figure 6). In the NPCCC group, two eyes experienced progression of PVD: one advanced from stage 2 to stage 4 at 1 month after surgery, and another progressed from stage 1 to stage 2 at 3 months postoperatively. In contrast, no progression of PVD was observed in the PPCCC group (Figure 7).

**Table 3:** Postoperative changes in PVD stage distribution.

PVD stage	PPCCC group	NPCCC group	*p*-value
Pre-op			
Stage 0	44 (74.58%)	41 (71.93%)	0.98
Stage 1	11 (18.64%)	11 (19.30%)	0.98
Stage 2	1 (1.69%)	2 (3.51%)	0.98
Stage 3	3 (5.08%)	2 (3.51%)	0.98
Stage 4	1 (1.67%)	1 (1.72%)	0.98
1 day			
Stage 0	44 (73.33%)	41 (71.93%)	0.98
Stage 1	11 (18.33%)	11 (19.30%)	0.98
Stage 2	1 (1.67%)	2 (3.51%)	0.98
Stage 3	3 (5.00%)	2 (3.51%)	0.98
Stage 4	1 (1.67%)	1 (1.72%)	0.98
1 week			
Stage 0	44 (73.33%)	41 (71.93%)	0.93
Stage 1	11 (18.33%)	11 (19.30%)	0.93
Stage 2	1 (1.67%)	2 (3.51%)	0.93
Stage 3	3 (5.00%)	2 (3.51%)	0.93
Stage 4	1 (1.67%)	1 (1.72%)	0.93
1 month			
Stage 0	44 (73.33%)	41 (71.93%)	
Stage 1	11 (18.33%)	11 (19.30%)	0.83
Stage 2	1 (1.67%)	1 (1.72%)	0.83
Stage 3	3 (5.00%)	2 (3.51%)	0.83
Stage 4	1 (1.67%)	2 (3.51%)	0.83
3 months			
Stage 0	44 (73.33%)	41 (71.93%)	0.81
Stage 1	11 (18.33%)	10 (17.54%)	0.81
Stage 2	1 (1.67%)	2 (3.51%)	0.81
Stage 3	3 (5.00%)	2 (3.51%)	0.81
Stage 4	1 (1.67%)	2 (3.51%)	0.81

Comparative variations in the number of different PVD stage at pre-surgery and at 1 day, 1 week, 1 month, and 3 months postoperatively between the two study groups.

## Discussion

Previous studies have evaluated posterior segment safety of cataract surgery with manual PPCCC (10, 11, 18, 19). However, there are some limitations, including the low resolution of OCT (TD-OCT), small sample size and lack of controlled group. Additionally, Schojai et al. (19) reported no postoperative CME in patients undergoing femtosecond laser-assisted PPCCC with SD-OCT, but the high cost and limited availability of femtosecond lasers, restrict its broader application and may limit the generalizability of the results. In this randomized controlled trial study, we employed SD-OCT to assess the changes in macular and PVD in patients with normal macula after cataract surgery combined with PPCCC over 3-months follow-up.

In our study, no statistically significant difference was found between the PPCCC group and NPCCC group in CRT, CSF, 1–3 mm middle ring and 3–6 mm outer rings preoperatively and at any postoperative visits. This result suggested that the macular thickness between cataract surgery with and without PPCCC is comparable. At 1 month postoperatively, we observed three eyes developed subtle CME (two were in NPCCC group, and one was in PPCCC group). Retinal thickness of one PPCCC eye and one NPCCC eye increased by 30% in the CRT, CSF, and 1–3 mm inner ring. Though the other NPCCC eye exhibited a significant 30% increase in CSF and 1–3 mm inner ring, the CRT did not show a 30% increase from baseline. The difference may be attributed to an early involvement of the parafoveal area (25). Past macular-related studies have largely focused on CRT and CSF (24, 26). However, prior scholars suggested that CME can also present as diffuse thickening, excluding parafoveal region might prone to false-negative results (22). In our study, we measured both point retinal thickness (CRT) and regional retinal thickness within a 6-mm area around the macula (including the CSF, 1–3 mm middle, and 3–6 mm outer rings). These parafoveal parameters may provide a more comprehensive assessment of macular thickness and help prevent underestimation of pathology (25).

In our study, after topical administration, the recovery time was 2 weeks for the PPCCC eye, compared to 8 weeks for the two NPCCC eyes. Previous studies reported that incomplete PVD progression could lead to vitreomacular traction syndrome, which is considered as a risk factor of CME (20, 21). It is important to noted that several studies have found that phacoemulsification may cause or accelerate previous PVD (27). However, the effect of combining PPCCC with cataract surgery on PVD is still unclear. In our study, we compared different PVD stage distributions preoperatively and at postoperative follow-ups. There are no significant differences in PVD progression between the PPCCC and NPCCC groups. Two eyes progressed PVD in the NPCCC group, and no PVD progression in the PPCCC group. This might suggest that cataract surgery performed with PPCCC does not increase the risk of vitreous macular traction and PCCC might associate with less PVD progression compared with traditional cataract surgery. A possible explanation for these observations is that PPCCC creates a window in the posterior capsule, allowing more surgical fluid flow into the Berger space through this opening (28). A study suggested that traditional cataract surgery process could result in the opening of the Berger space (29). We hypothesize that PPCCC allows even more fluid flow into the Berger space, this not only results in a wider burger space but also causes the anterior vitreous surface to further detach from the central posterior capsule. This detachment could potentially weaken anteriorly oriented traction forces due to postoperative capsular fibrosis and facilitate the vitreous collapsing more backward (11). This may also explain why the CME eye in the PPCCC group had a shorter recovery time.

Furthermore, in our study, we included 22 diabetic patients with normal macula and found no CME cases in both groups postoperatively. Different with our result, Jukić et al. (23) has reported that the CME incidence is 22.0% in DM patients after cataract surgery. We assume that this difference is because Kim et al. (30) included DM patients with diabetic retinopathy (DR), whereas our study excluded. Moreover, it is known that DM patients have a higher prevalence of early-onset cataracts and experience more rapid formation of PCO, which necessitates early cataract surgery and subsequent postoperative Nd:YAG laser capsulotomy (31, 32). This population is more likely benefit from PPCCC. Notably, compared to non-diabetic patients, DM patients without diabetic retinopathy (DR) exhibit a potentially 1.8-fold elevated risk of CME following cataract surgery (24). However, whether PPCCC further exacerbates this risk in this specific population remains unclear and is needed to further investigate (24). Though prior researchers had evaluated the macula-related safety of PPCCC, they generally excluded DM patients (10, 17–19). In our study, we assessed the safety of PPCCC specifically for the posterior segment of the eye in early DM patients without DR. There are also no significant changes in retinal thickness in early DM patients without DR between two groups at any scheduled visit. This may suggest that PPCCC does not elevate the risk of CME and can be safely utilized in DM patients with a normal macula.

The strength of this randomized clinical trial is the prospective, intraindividual randomized controlled trial design which brings convincing clinical evidence. Furthermore, we extend the macular thickness evaluation, broadening the macular thickness from horizontal CRT, CSF to the 1–3 mm middle and 3–6 mm outer rings. Lastly, we included patients with preoperative PVD and DM, and further analyzed the posterior segment safety of PPCCC for these population, which might broaden the application of PPCCC.

The present study has several limitations. First, as this is a single center randomized controlled trial involving 65 patients, a multicenter trial with larger sample size may be necessary to validate our findings. Second, we only observed for 3 months. Though based on previous studies showing that CME typically peaks around 5–6 weeks postoperatively (24), a longer follow-up should be required to assess long-term outcomes in future study. Third, the surgeon could not be masked to the intervention during the procedure, which could introduce bias. However, as the surgeon was not involved in subsequent evaluations or data analysis, the potential for bias in the randomization process is mitigate.

In conclusion, cataract surgery with stand-alone PPCCC does not increase the risk of postoperative CME and PVD progression in patients with a normal macula, including those with DM. PPCCC can be considered a safe cataract surgery technique option.

## Data Availability

The original contributions presented in the study are included in the article/Supplementary material, further inquiries can be directed to the corresponding author.
