# Supplementary material for: Macular morphology after cataract surgery with and without primary posterior continuous curvilinear capsulorhexis
Source: Front Med (Lausanne). 2025 Oct 28;12:1687460. doi: 10.3389/fmed.2025.1687460 (PMC12602534; doi:10.3389/fmed.2025.1687460)
Supplement: Supplementary file 1 [file Table_1.DOCX]

**Supplement data**

**Table 2.** CDVA, CRT, CSF, averaged retinal thickness of 1-3mm middle and 3-6mm outer rings.

| Parameters | PPCCC group | NPCCC group | P-value |
| --- | --- | --- | --- |
| CDVA (logMAR) |  | | |
| 1d | 0.2±0.18 | 0.23±0.16 | 0.23 |
| 1wk | 0.06±0.08 | 0.05±0.07 | 0.49 |
| 1mo | 0.03±0.06 | 0.03±0.06 | 0.75 |
| 3mo | 0.02±0.04 | 0.01±0.03 | 0.63 |
| CRT |  | | |
| 1d | 215.8±15.8 | 216.2±16.0 | 0.90 |
| 1wk | 213.2±15.7 | 215.4±16.6 | 0.46 |
| 1mo | 222.6±21.8 | 227.2±25.9 | 0.29 |
| 3mo | 222.0±17.3 | 223.9±16.5 | 0.54 |
| CSF |  | | |
| 1d | 250.2±13.2 | 250.98±15.3 | 0.75 |
| 1wk | 251.6±14.6 | 251.8±16.4 | 0.95 |
| 1mo | 262.5±19.8 | 264.8±26.7 | 0.59 |
| 3mo | 258.8±16.3 | 259.1±17.8 | 0.92 |
| 1-3mm area |  |  |  |
| 1d | 320.1±13.3 | 321.3±14.0 | 0.61 |
| 1wk | 325.7±13.5 | 325.7±15.4 | 0.97 |
| 1mo | 330.8±15.7 | 332.1±19.3 | 0.69 |
| 3mo | 330.4±13.5 | 331.8±15.0 | 0.90 |
| 3-6mm area |  |  |  |
| 1d | 285.0±14.5 | 285.9±14.2 | 0.75 |
| 1wk | 288.8±13.7 | 288.3±14.5 | 0.86 |
| 1mo | 293.9±15.0 | 294.6±15.3 | 0.82 |
| 3mo | 293.7±14.3 | 292.7±15.7 | 0.87 |

The CDVA, CRT, CSF, averaged retinal thickness of 1-3mm middle and 3-6mm outer rings change between PPCCC group and NPCCC group at 1 day,1week, 1month, and 3months postoperatively.

**Table 3. Postoperative Changes in PVD Stage Distribution**

| PVD stage | PPCCC group | NPCCC group | P-value |
| --- | --- | --- | --- |
| pre-op |  | | |
| stage 0 | 44(74.58%) | 41(71.93%) | 0.98 |
| stage 1 | 11(18.64%) | 11(19.30%) | 0.98 |
| stage 2 | 1(1.69%) | 2(3.51%) | 0.98 |
| stage 3 | 3(5.08%) | 2(3.51%) | 0.98 |
| stage 4 | 1(1.67%) | 1(1.72%) | 0.98 |
| 1 day |  | | |
| stage 0 | 44(73.33%) | 41(71.93%) | 0.98 |
| stage 1 | 11(18.33%) | 11(19.30%) | 0.98 |
| stage 2 | 1(1.67%) | 2(3.51%) | 0.98 |
| stage 3 | 3(5.00%) | 2(3.51%) | 0.98 |
| stage 4 | 1(1.67%) | 1(1.72%) | 0.98 |
| 1 week |  | | |
| stage 0 | 44(73.33%) | 41(71.93%) | 0.93 |
| stage 1 | 11(18.33%) | 11(19.30%) | 0.93 |
| stage 2 | 1(1.67%) | 2(3.51%) | 0.93 |
| stage 3 | 3(5.00%) | 2(3.51%) | 0.93 |
| stage 4 | 1(1.67%) | 1(1.72%) | 0.93 |
| 1 month |  |  |  |
| stage 0 | 44(73.33%) | 41(71.93%) |  |
| stage 1 | 11(18.33%) | 11(19.30%) | 0.83 |
| stage 2 | 1(1.67%) | 1(1.72%) | 0.83 |
| stage 3 | 3(5.00%) | 2(3.51%) | 0.83 |
| stage 4 | 1(1.67%) | 2(3.51%) | 0.83 |
| 3 months |  |  |  |
| stage 0 | 44(73.33%) | 41(71.93%) | 0.81 |
| stage 1 | 11(18.33%) | 10(17.54%) | 0.81 |
| stage 2 | 1(1.67%) | 2(3.51%) | 0.81 |
| stage 3 | 3(5.00%) | 2(3.51%) | 0.81 |
| stage 4 | 1(1.67%) | 2(3.51%) | 0.81 |

Comparative variations in the number of different PVD stage at pre-surgery and at 1 day, 1 week, 1 month, and 3 months postoperatively between the two study groups.
