# Supplementary material for: Macular morphology after cataract surgery with and without primary posterior continuous curvilinear capsulorhexis
Source: Front Med (Lausanne). 2025 Oct 28;12:1687460. doi: 10.3389/fmed.2025.1687460 (PMC12602534; doi:10.3389/fmed.2025.1687460)
Supplement: Supplementary file 2 [file Table_2.DOCX]

**Supplementary materials:** Sensitivity analysis using all three threshold definitions

|  | PPCCC group | NPCCC group | ***P* value** |
| --- | --- | --- | --- |
| 110% baseline CRT | 1.67% (1/60) | 11.67% (7/60) | 0.07 |
| 130% baseline CRT | 1.67% (1/60) | 3.33% (2/60) | ＞0.99 |
| 140% baseline CRT | 1.67% (1/60) | 3.33% (2/60) | ＞0.99 |
